# Effects of Periodic Task-Specific Test Feedback on Physical Performance in Older Adults Undertaking Band-Based Resistance Exercise

**DOI:** 10.1155/2014/171694

**Published:** 2014-01-29

**Authors:** Ryuichi Hasegawa, Mohammod Monirul Islam, Ryuji Watanabe, Naoki Tomiyama, Dennis R. Taaffe

**Affiliations:** ^1^Department of Occupational Therapy, College of Life and Health Sciences, Chubu University, 1200, Matsumoto, Kasugai, Aichi 487-8501, Japan; ^2^Department of Rehabilitation, Yonaha General Hospital, 8-264-3 Izumi, Kuwana, Mie 511-0838, Japan; ^3^Department of Rehabilitation, Tsushima City Hospital, 3-73 Tsubaki, Tsushima, Aichi 496-8537, Japan; ^4^Division of Occupational Therapy, Faculty of Care and Rehabilitation, Seijoh University, 2-172 Fukinodai, Tokai, Aichi 476-8588, Japan; ^5^School of Environmental and Life Sciences, The University of Newcastle, 10 Chittaway Road, Ourimbah, NSW 2258, Australia

## Abstract

The purpose of this study was to determine the effects of periodic task-specific test feedback on performance improvement in older adults undertaking community- and home-based resistance exercises (CHBRE). Fifty-two older adults (65–83 years) were assigned to a muscular perfsormance feedback group (MPG, *n* = 32) or a functional mobility feedback group (FMG, *n* = 20). Both groups received exactly the same 9-week CHBRE program comprising one community-based and two home-based sessions per week. Muscle performance included arm curls and chair stands in 30 seconds, while functional mobility was determined by the timed up and go (TUG) test. MPG received fortnightly test feedback only on muscle performance and FMG received feedback only on the TUG. Following training, there was a significant (*P* < 0.05) interaction for all performance tests with MPG improving more for the arm curls (MPG 31.4%, FMG 15.9%) and chair stands (MPG 33.7%, FMG 24.9%) while FMG improved more for the TUG (MPG-3.5%, FMG-9.7%). Results from this nonrandomized study suggest that periodic test feedback during resistance training may enhance task-specific physical performance in older persons, thereby augmenting reserve capacity or potentially reducing the time required to recover functional abilities.

## 1. Introduction

Functional fitness not only accounts for the traditional physical fitness parameters such as muscle strength, cardiorespiratory endurance, and flexibility, but also includes balance [1]. However, it is well known that, with age, functional fitness declines [2], resulting in a reduced ability to perform activities such as rising from a chair or climbing stairs, which can be viewed as functional components of activities of daily living (ADL) performance, and may eventually compromise the ability to perform ADL [3–5]. In addition, approximately a third of community-dwelling older adults fall at least once a year, and over 30% of fallers suffer injuries requiring medical attention [6]. Strategies to prevent or attenuate the decline in physical function and balance are important for promoting independence in the elderly and thereby enhancing quality of life.

Research over the past two decades clearly shows that regular physical exercise is effective for maintaining and promoting health, physical fitness, and functional independence in older adults, especially in terms of endurance, muscular strength, flexibility, and balance [7]. Physical training programs can be undertaken in the clinic, commercial fitness centers or gymnasiums, in the community or at home. In contrast to clinic and gym-based exercise, community- and home-based exercise does not require special facilities or expensive equipment. Moreover, community- and home-based exercise is capable of reaching a broad audience and, importantly, the community setting provides an opportunity for social contact and support which may have an array of benefits, especially for those living in regional and rural settings [8]. In order to maximize compliance to community- and home-based programs, exercises should be simple, combined with appropriate equipment and personal support. Thus, community- and home-based exercises have become increasingly popular as an alternative to gym-based resistance exercise [9].

Providing feedback and prompting have been shown to be effective in increasing and maintaining physical activity as well as other positive health behaviors [10]. Moreover, feedback may also enhance the exercise response which may prove valuable in enhancing recovery of functional abilities after injury or illness [11, 12]. However, a paucity of information is currently available regarding the effects of feedback on muscular performance and functional mobility improvement in older adults undertaking resistance training in the community- and home-based setting. Hasegawa and Tomiyama have previously reported, that in middle-aged and older persons participating in a community- and home-based setting, periodic feedback enhanced muscle performance compared to those not receiving feedback [13]. Consequently, the purpose of this study was to confirm and extend these findings by examining the effects of periodic task-specific test feedback on not only muscle performance but also functional mobility in older adults undertaking resistance training with elastic bands in the community- and home-based setting.

## 2. Materials and Methods

### 2.1. Subjects

In response to a public relations magazine advertisement, 80 older adults from Tokai city, Aichi prefecture, Japan, volunteered to participate in the study. Inclusion criteria were ≥65 years of age, community residing, functionally independent, and able to perform physical exercise. Participants were excluded if they had been advised by their physician to refrain from exercise. Prior to acceptance into the study, a brief health examination was performed by an occupational therapist and questionnaires regarding medical history were completed. Current presence of disease was determined using self-reported physician-diagnosed disease information. Two persons were found medically unfit due to uncontrolled hypertension and cardiac arrhythmia. Three others were excluded from the study because they had scheduling conflicts, transportation issues, or no further interest in joining the study.

The remaining 75 participants (65 to 83 years) were included in this study. They were divided into a muscular performance feedback group (MPG) or a functional mobility feedback group (FMG) by their residential area (the two groups were from two separate communities within the same city). The two residential areas had similar living standards and socioeconomic status. Both groups participated in an identical 9-week community- and home-based resistance exercise program. All participants were asked to not alter their diet or physical activity patterns for the duration of the study. All measurements were performed before and after the 9-week program in 52 participants. Twenty-three participants did not complete follow-up measures due to travel-related reasons (*n* = 18) and medically-related problems (low back pain, *n* = 2; dizziness, *n* = 1; upper respiratory tract infection, *n* = 2). Regarding baseline characteristics, there was no difference between those who dropped out and those who completed the study within each group for age, weight or any functional fitness measure, with the only difference being in the MPG with more women than men dropping out (due to travel) and hence height of those who dropped out was lower than those who completed.

**Figure 1:**
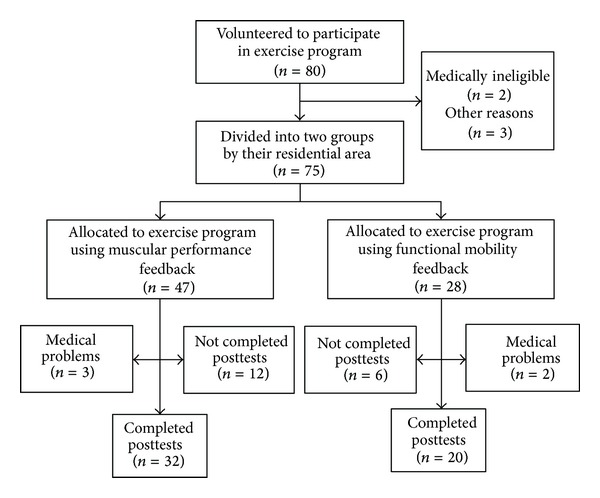
Flowchart tracking participants throughout the trial.

A flowchart of the study participants is provided in Figure 1. The ethics committee of Seijoh University approved the study and all participants provided written informed consent.

### 2.2. Exercise Program

Instruction and progression of the exercise routine occurred at a community center once per week and participants were asked to exercise an additional two times each week at home. The community-based exercise sessions consisted of 15 min of warmup, 60 min of resistance exercise, and 15 min of cooldown. The exercise sessions were supervised by experienced instructors. Pictorial guidebooks were provided to all participants in order to assist them to perform the exercises correctly. The home-based exercise program also consisted of the same resistance and stretching exercises. Participants were asked to record exercises they performed and submit a diary every week while attending the exercise classes.

#### 2.2.1. Resistance Exercise

**Figure 2:**
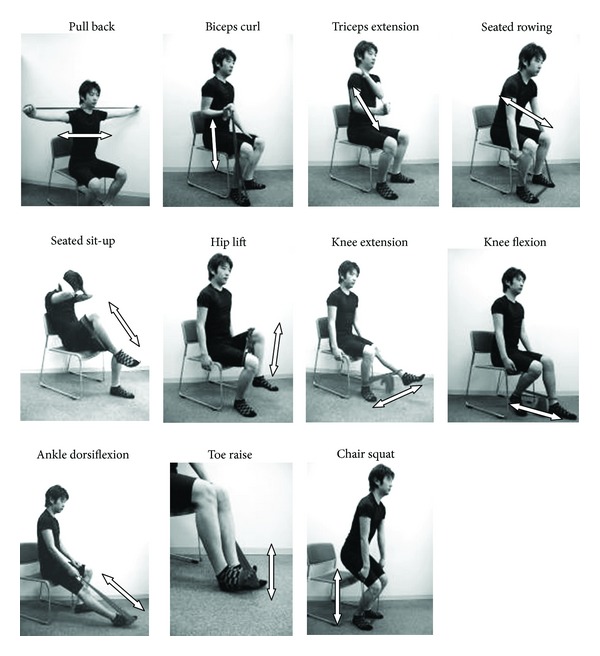
Resistance exercises undertaken in the community- and home-based setting. The resistance training program consisted of exercises that used an elastic resistance band (Thera-Band, Hygenic, USA). Each type of exercise was performed for 12 repetitions/session. Exertion was rated using Borg's rate of perceived exertion (RPE) scale.

In order to train all major muscle groups, resistance exercises were prescribed as a combination of 3 upper body exercises, 6 lower body exercises, and 2 trunk exercises performed using an elastic resistance band (Thera-Band, Hygenic, USA). Each type of exercise was performed for 1 set of 12 repetitions per session [14]. Exertion was rated using Borg's rate of perceived exertion (RPE) scale [15, 16]. Participants were instructed to start resistance exercises at an intensity level of 13 on the RPE scale and then to progressively increase resistance to a level of 15 to 17. Participants were instructed to progressively increase resistance every two to four weeks by advancing to the next color of elastic band (lower to higher resistance of bands in order: red, green, and blue) or shortening the initial length of the band for increased resistance (Figure 2).

#### 2.2.2. Stretching Exercise as Warmup and Cooldown

Stretching exercises consisted of eight upper body exercises and seven lower body exercises. The exercises were performed slowly and each position was held for 20 seconds. The participants were asked to stretch to the point where they felt moderate tension without feeling pain in joints or muscles.

### 2.3. Feedback

Fortnightly assessments were performed by testers for three performance tests: arm curls, chair stands in 30 seconds, and the timed up and go (TUG) test. Task specific feedback, which is extrinsic or augmented information provided to a performer in regard to a specific task with the goal to enhance future performance of that task [17], was provided by the testers (not instructors) who were blinded to the participant's previous results. MPG received immediate test feedback (verbal and written) only on the arm curls and chair stand test while FMG received immediate feedback only for the TUG. Participants recorded their results on individual record sheets. In addition to the participant's results for the respective tests (muscle performance or balance), verbal feedback from the tester included statements (in front of other participants) such as “you have improved in your performance and it shows in your current score” and “keep up the exercise and you are going to improve your performance.” All measurements were performed by the same tester.

### 2.4. Study Measures

Anthropometrics, physical symptoms by questionnaire, muscle performance, and functional mobility were evaluated at baseline and followup. Participant's height and weight were assessed and body mass index (BMI) was calculated as body weight (kg) divided by the square of height (m).

#### 2.4.1. Measurement of Muscular Performance

Upper body muscle performance was assessed using the 30-second arm curl test (arm curl) [18, 19]. On a signal, participants were instructed to flex and extend the elbow of the dominant hand, lifting a weight dumbbell (men: 8-lbs [3.6 kg], women: 5-lbs [2.3 kg]) through the complete range of motion, as many times as possible in 30 seconds. A practice trial of one or two repetitions was given, followed by two test trials. The score was the number of repetitions completed with the best performance used for analysis.

Lower body muscle performance was assessed using the 30-second chair stand test [18, 19]. The participant's arms were crossed at the wrists and held against the chest. On a signal, participants rose to a standing position from a chair and then returned to a seated position and continued to complete as many full stands as possible in 30 seconds. A practice trial of one or two repetitions was given, followed by two test trials. The score was the total number of stands executed correctly with the best performance used for analysis.

#### 2.4.2. Measurement of Functional Mobility

Functional mobility was assessed using the 8-foot timed up and go (TUG) test [18–20]. Participants sat in a chair with their hands on their thighs and feet flat on the floor. On a signal, participants stood from the chair without pushing off with the arms, walked as quickly as possible around a cone placed 8-feet (2.44 m) ahead of the chair, and returned to a fully seated position in the chair. Participants were instructed to walk as quickly as possible without running. Participants walked through the test one time as a practice and then were given two test trials with the best performance time used for analysis.

### 2.5. Statistical Analysis

Data were analyzed using the PASW statistics 18 software package (SPSS Inc., Chicago, IL, USA). Comparisons between MPG and FMG at baseline were performed using an independent Student's *t*-test or Chi-square test as appropriate. The effect of the intervention was determined using a repeated measures analysis of covariance (ANCOVA) adjusted for gender and within group changes by a paired *t*-test. All tests were two tailed and a *P* value of less than 0.05 was considered statistically significant. Values reported are the mean ± SD.

## 3. Results

### 3.1. Pretraining Data

There were no differences between groups at baseline for age, height, weight, and prevalent disease (Table 1) or muscle performance and functional mobility (Table 2).

### 3.2. Training Data

The average adherence rates in the exercise class at the community center were 92 ± 13% for MPG and 87 ± 12% for FMG. Both groups performed home-based exercises in addition to community-based classes for a total of 2.7 ± 1.3 days/week in MPG and 2.1 ± 1.0 days/week in FMG. No differences were observed between the two groups in adherence or home-based training frequency (Table 1). There were no accidents or injuries during the exercise classes at the community center or at home.

There was a significant group × time interaction for the arm curl (*F* = 15.2) and chair stand test (*F* = 5.2) with MPG improving more than FMG, while FMG improved more than MPG for the TUG (*F* = 4.1) (Table 2). In the MPG, the muscle performance measures for the arm curl and chair stand test increased by 31.4% (*P* = 0.001) and 33.7% (*P* < 0.001), respectively, while TUG performance time was reduced by 3.5% (*P* < 0.001). In the FMG, arm curl and chair stand test significantly increased by 15.9% (*P* < 0.001) and 24.9% (*P* < 0.001), respectively, while time to perform the TUG was reduced by 9.7% (*P* = 0.049).

## 4. Discussion

Maintaining or enhancing muscle performance and functional mobility in older persons is critical for undertaking daily activities and for sustaining an appropriate quality of life. In the current study, we found significant improvements in muscle performance and functional mobility following a 9-week community- and home-based exercise regimen undertaken with elastic bands. Importantly, gains were partly dependent on fortnightly test feedback suggesting that this is an important component of the training session in order to maximize gains resulting from the program.

The muscular performance results are in agreement with other studies that have used elastic bands in a community setting. Bohlken et al. reported significant increases of 16% in arm curl and 18% in chair stand performance in older women who participated in a 12-week exercise program (3 d/wk) using elastic bands [21]. Similarly, a 23% and 19% increase in arm curl and chair stand performance, respectively, was reported by Rogers et al. who enrolled older women in a 4-week (3 d/wk) elastic band resistance exercise program [22]. However, gains resulting from home-based programs may not be of the same magnitude as that of supervised gym- or community-based programs with Yamauchi et al. reporting improvements of 18% in the arm curl test and only 6% in the chair stand test for older adults following 12 weeks of a home-based exercise program [9].

In the current study, the FMG had an average improvement for the arm curl test of 16%, which is comparable to other studies with a similar training period. However, the MPG improvement on this task was double that of the FMG with gains of 31%. These results suggest that task-specific feedback of serial testing may be an effective strategy to enhance performance in older persons. For the chair stand test, there was also a significant difference between the two feedback groups with the task-specific group enhancing their performance by ~34% while the FMG increased by ~25%. Moreover, the results in MPG were higher than previous studies with comparable training programs [9, 21, 22]. The improvements in the chair stand test are particularly important given the role that lower body muscle performance, as does balance, plays in maintaining physical function.

Similarly, for the timed up and go test, which was used to assess functional mobility in our cohort, improvement was greater for the FMG who decreased their performance time by ~10% whereas the MPG experienced a reduction of only 3.5%. This suggests that improvements in functional mobility are also associated with the use of feedback in older persons.

These results indicate that the magnitude of improvement in muscular function and functional mobility are associated with the feedback provided and would be beneficial to older persons in order to maximize program-related gains. The importance of data feedback has been previously described by Mihalko et al. who reported that individual fitness feedback influences exercise attendance [23]. Positive feedback is effective in intensifying competence [24]. In addition, it is suggested that relatedness is intensified in group work and exercise programs in which the participant's interaction is increased [25]. Moreover, Bourbonnais et al. reported that treatment of the lower limb in stroke patients based on muscle force-feedback produced an improvement in gait velocity [26], while feedback has also been found to improve pelvic floor muscle training in those with urinary incontinence [27]. In athletes, providing verbal feedback has been associated with a modest enhancement in muscle performance [28] and tuck-jump performance [29], although not in time trial cycling [30]. Our results reinforce the importance of providing feedback if serial performance assessments are undertaken.

Feedback can be classified as either “intrinsic” or “extrinsic” with task-specific feedback categorized as knowledge of results [16]. Indeed, the observations of Leventthal have documented the importance of providing relevant information in order to encourage an action [31]. It may well be that awareness of muscular performance or functional mobility improvement and the encouragement provided by the tester (in delivering the feedback in a face-to-face situation) enhanced the participant's self-efficacy [32] for these respective tasks and their intrinsic motivation [33, 34] and this contributed to task-specific performance differences in the MPG and FMG. In addition, the feedback may have reinforced or enhanced the participant's goal-setting which in turn enhanced their intrinsic motivation [35]. The finding that feedback of performance may have increased motivation is consistent with previous literature and cognitive evaluation theory [36] which proposes that, when feelings of competence are enhanced, motivation increases.

Motivating older adults to perform exercises on a regular basis is an important factor in maintaining the effects of exercise [9]. In the current study, we held community-based exercise classes once a week in addition to the home-based exercise program 2 days per week. The adherence to the community-based classes was over 85% and the frequency of the community- and home-based exercise was over 2 days per week. This finding of frequency meets the current guidelines for resistance exercise [12]. Most participants mentioned that they were glad to make new friends and enjoyed exercising as a group in the community setting. This supports previous findings [37] that community-based exercise classes have positive psychological effects as well as physical effects on community-dwelling older adults. After completion of this 9-week program, participants continued to exercise and currently attend exercise classes twice a month. Enhanced self-efficacy resulting from participation may be one factor that contributed to the participation rates during the study period as well as with the participation postintervention [32].

There are several limitations of the study which are worthy of comment. Given the homogeneity of participants in the study (age, socioeconomic status, and geographical location), caution should be taken when extrapolating our results to all older community-dwelling persons. Moreover, the participants were not randomized to the treatment conditions as it was not possible to blind participants to the exercise program, leaving them vulnerable to a variety of tester and subject effects that may influence test results and introduce bias in our findings. Our program was also of a relatively short-term nature and it is unclear if differences based on feedback would exist with a longer program, such as 6 or 12 months. Nevertheless, our results show, even within a relatively short training period, that task-specific test feedback appears to have a beneficial effect in enhancing physical performance, and, regardless of the underlying mechanism for the improvement, this is an important practical outcome for those involved in the exercise training of older persons. A future direction would be in determining if task-specific feedback is a valuable strategy to enhance performance in those disabled or recovering from illness/disease or injury. In addition, a more extensive test battery could be employed to measure additional aspects of functional and ambulatory ability. Lastly, it would be of interest to determine if performance was enhanced by motivation, whether it was motivation to train harder or to perform the tests or to the combination.

## 5. Conclusion

This study was designed to determine the efficacy of test performance feedback to improve muscle performance and functional mobility in older adults. Following the 9-week community- and home-based intervention, a significant interaction was noted for the muscle performance and functional mobility tests suggesting that periodic test feedback during resistance training may enhance task-specific physical performance in older persons. Providing regular feedback on test performance in the community and, if possible, the home exercise setting may facilitate gains in muscle and balance performance resulting in enhanced physical function and a greater safety margin for functional thresholds.

## Figures and Tables

**Table 1:** Participant characteristics (mean ± SD).

Variables	Muscle performance feedback group	Functional mobility feedback group	*P* values
No. of participants	32	20	
Demographic			
Gender (men/women)^†^	16/16	5/15	0.09
Age (years)^‡^	71.4 ± 4.3	73.7 ± 5.5	0.10
Anthropometrics			
Height (cm)^‡^	155.4 ± 6.8	152.1 ± 7.5	0.11
Weight (kg)^‡^	56.7 ± 9.0	55.5 ± 8.5	0.63
BMI (kg/m^2^)^‡^	23.4 ± 3.0	24.0 ± 3.2	0.53
Prevalent disease [number (%)]			
Hypertension^†^	8 (25%)	9 (45%)	0.14
Knee osteoarthritis^†^	8 (25%)	5 (25%)	1.00
Diabetes mellitus^†^	4 (13%)	3 (15%)	0.55
Heart disease^†^	4 (13%)	3 (15%)	0.55
Osteoporosis^†^	2 (6%)	1 (5%)	0.67
Compliance of exercise			
Adherence at community center (%)^‡^	92.0 ± 13.0	87.2 ± 12.1	0.11
Frequency (days/week)^‡^	2.7 ± 1.3	2.1 ± 1.0	0.19

Note: BMI: body mass index.

No significant differences at baseline were present between groups for all indexes.

^†^ *χ* ^2^ test was used to evaluate differences between the groups.

^‡^Student's *t*-test was used to evaluate the difference between the groups.

**Table 2:** Improvements in muscle performance and functional mobility.

	Muscle performance feedback group			Functional mobility feedback group			Interaction (*F* ratio)
	Pre	Post	% Change	Pre	Post	% Change	
Muscle performance							
Arm curl (reps/30 sec.)	21.6 ± 2.7	28.4 ± 3.6	31.4%^§^	20.7 ± 3.0	24.0 ± 3.7	15.9%^§^	15.2*
Chair stand (reps/30 sec.)	20.2 ± 2.4	27.0 ± 4.2	33.7%^§^	18.9 ± 5.1	23.6 ± 5.1	24.9%^§^	5.2*
Functional mobility							
Timed up & go (sec)^#^	4.87 ± 0.57	4.70 ± 0.50	−3.5%^§^	5.18 ± 1.00	4.68 ± 0.78	−9.7%^§^	4.1*

Note: Values are mean ± SD.

Pre: baseline; post: after the 9-week exercise program.

No significant differences at baseline were present between groups for all indexes.

^#^Improvement on test results in a negative value.

*Analysis of covariance (ANCOVA) significant group by time interaction, *P* < 0.05.

^§^Paired *t*-test, *P* < 0.05.
